# Venoarterial extracorporeal membrane oxygenation for group B streptococcal toxic shock syndrome: A case report and literature review

**DOI:** 10.1097/MD.0000000000034680

**Published:** 2023-09-15

**Authors:** Naoya Iwasaki, Motohiro Sekino, Tetsuro Tominaga, Takeshi Tanaka, Hiroshi Araki, Rintaro Yano, Sojiro Matsumoto, Taiga Ichinomiya, Ushio Higashijima, Takashi Nonaka, Koichi Izumikawa, Tetsuya Hara

**Affiliations:** a Department of Anesthesiology and Intensive Care Medicine, Nagasaki University Graduate School of Biomedical Sciences, Nagasaki, Japan; b Department of Surgical Oncology, Nagasaki University Graduate School of Biomedical Sciences, Nagasaki, Japan; c Infection Control and Education Center, Nagasaki University Hospital, Nagasaki, Japan.

**Keywords:** cardiopulmonary arrest, ECMO, mortality, septic shock, streptococcal infections, *Streptococcus agalactiae*, STSS

## Abstract

**Rationale:**

Streptococcal toxic shock syndrome (STSS) rapidly leads to refractory shock and multiple organ failure. The mortality rate among patients with STSS is 40%; however, most deaths occur within a few days of onset. Venoarterial extracorporeal membrane oxygenation (VA-ECMO) may help avoid acute death in adult patients with STSS. However, the effectiveness of VA-ECMO is unclear. In this study, we report a case of group B STSS, which was successfully treated with VA-ECMO despite cardiopulmonary arrest (CPA) owing to rapidly progressive refractory shock.

**Patient concerns:**

A 60-year-old woman was hospitalized because of diarrhea and electrolyte abnormalities owing to chemoradiation therapy for rectal cancer. A sudden deterioration of her condition led to CPA. Conventional cardiopulmonary resuscitation was immediately performed but was ineffective. Therefore, VA-ECMO was initiated. Contrast-enhanced computed tomography revealed duodenal perforation. Hence, septic shock owing to peritonitis was diagnosed, and emergency surgery was performed under VA-ECMO. However, the patient had progressive multiple organ failure and required organ support therapy in the intensive care unit (ICU).

**Diagnoses:**

On day 2 in the ICU, blood and ascites fluid culture tests revealed beta-hemolytic streptococci, and the patient was finally diagnosed as having STSS caused by *Streptococcus agalactiae*.

**Interventions:**

Clindamycin was added to meropenem, vancomycin, and micafungin, which had been administered since the sudden deterioration. In addition, VA-ECMO, mechanical ventilation, blood purification therapy, and treatment for disseminated intravascular coagulation were continued.

**Outcomes:**

Thereafter, hemodynamics improved rapidly, and the patient was weaned off VA-ECMO on day 5 of ICU admission. She was transferred to a general ward on day 22 in the ICU.

**Lessons:**

In patients with fatal STSS and rapid progressive refractory shock or CPA, VA-ECMO may help to avoid acute death and improve prognosis by ameliorating tissue oxygenation and providing extra time to treat invasive streptococcal infection.

## 1. Introduction

Streptococcal toxic shock syndrome (STSS) is a relatively rare disease with a poor prognosis. It rapidly leads to shock and multiple organ failure among patients, with a reported mortality rate of approximately 40% and most patients dying within days of onset. ^[1,2]^STSS is typically caused by group A *Streptococcus* (GAS; *S. pyogenes*) but may also be caused by other groups of beta-hemolytic streptococci. Recently, group B *Streptococcus* (GBS; *S. agalacticae*) in nonpregnant adults and group G *Streptococcus* have been increasingly reported as the causative organisms, with mortality rates as high as those due to STSS caused by GAS.^[3–6]^ The mechanism of STSS is not completely understood; however, it has been linked to a combination of effects of streptococcal super antigenic toxins, other streptococcal enzymes and toxins, and host response to streptococcal infection.^[2]^ Early diagnosis and multidisciplinary management, including intensive care for multiple organ failure, control of the source of infection, surgical management, and the administration of antibiotics, including clindamycin for inhibition of toxin production, are critical for patients with STSS.^[2]^

The health of some patients with STSS, including otherwise healthy individuals with no comorbidities, may deteriorate rapidly after the onset of STSS, leading to refractory shock and finally cardiopulmonary arrest (CPA). In recent years, several case reports on adult patients with STSS who were introduced to venoarterial extracorporeal membrane oxygenation (VA-ECMO) have been published.^[7–13]^ However, the effectiveness of VA-ECMO for septic shock in adults is controversial.^[14–17]^ In patients aged 60 years or older,^[14]^ distributive shock with normal cardiac function^[17]^ and CPA before ECMO initiation^[18]^ have been reported as poor prognostic factors of ECMO therapy for septic shock. However, whether VA-ECMO plays a role in improving the prognosis of patients with STSS remains unknown.

Thus, herein we report a case of group B STSS, which was successfully treated with VA-ECMO despite CPA owing to rapidly progressive refractory shock.

## 2. Case report

A 60-year-old woman (height, 164 cm; weight, 51.6 kg) was hospitalized because of diarrhea and electrolyte abnormality owing to chemoradiotherapy for rectal cancer. She had a medical history of hypertension. Her general condition gradually improved after admission; her hemodynamic and respiratory statuses were stable, and signs of infection such as fever were absent.

**Figure 1.**
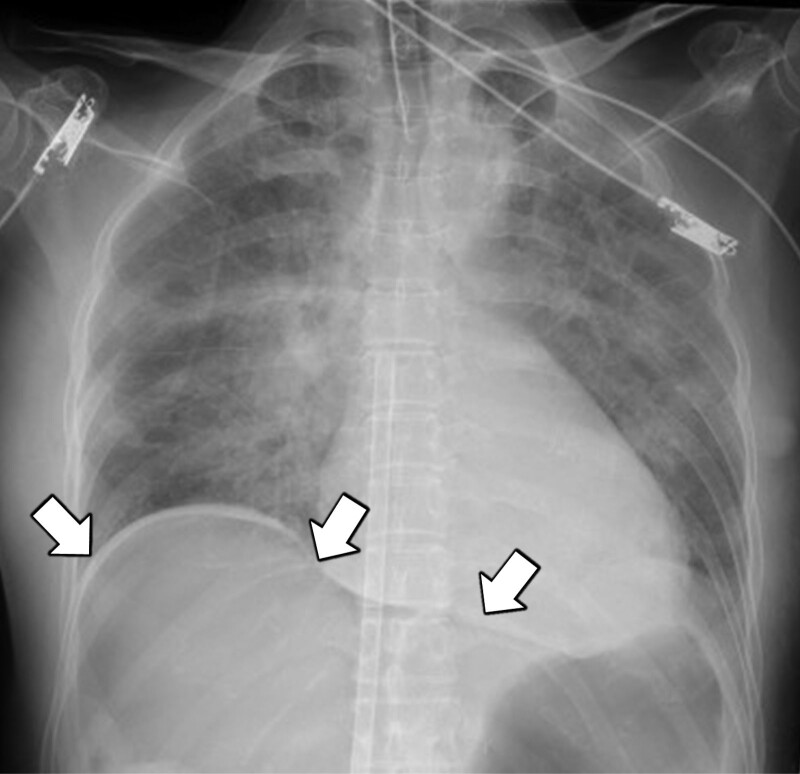
Chest radiograph obtained after VA-ECMO induction showing diffuse infiltrative shadows suggestive of acute respiratory distress syndrome and free air in the abdominal cavity (white arrows). VA-ECMO = venoarterial extracorporeal membrane oxygenation.

However, on day 7 of hospitalization, she experienced slight disorientation followed by somnolence. She was tachycardic, with a heart rate of 120 bpm, but stable blood pressure at 119/73 mm Hg and body temperature of 36.9°C. Due to decreased oxygenation, nasal oxygen at 2 L/minutes was started, and she was kept under observation. Early in the morning of day 8 of hospitalization, she was suddenly comatose and had a fever (body temperature, 39.8°C). She had refractory shock, which rapidly progressed to CPA with pulseless electrical activity. Conventional cardiopulmonary resuscitation (CPR) was immediately performed. An electrocardiogram obtained during CPR showed asystole or pulseless electrical activity. CPR, including 7 infusions of 1 mg intravenous adrenaline, failed to achieve return of spontaneous circulation (ROSC). Therefore, VA-ECMO was introduced 38 minutes after initiating CPR. A venous drainage cannula (21 Fr) and arterial return cannula (16.5 Fr) (both CAPIOX, percutaneous catheter, Terumo, Tokyo, Japan) for ECMO were inserted into the right femoral vessels. ECMO was maintained with blood flow at 3.0 L/minutes using a centrifugal pump (CAPIOX emergency bypass system, Terumo, Tokyo, Japan) and a membrane oxygenator (CAPIOX LX, Terumo, Tokyo, Japan). ROSC was achieved briefly after initiating VA-ECMO. Blood biochemistry analyses performed at CPA showed hyperkalemia (potassium level, 7.1 mmol/L), which was presumed to be one of the causes of cardiac arrest. A chest radiograph showed bilateral diffuse infiltrative shadows, leading to the suspicion of acute respiratory distress syndrome (ARDS), and free air in the abdominal cavity (Fig. 1).

**Figure 2.**
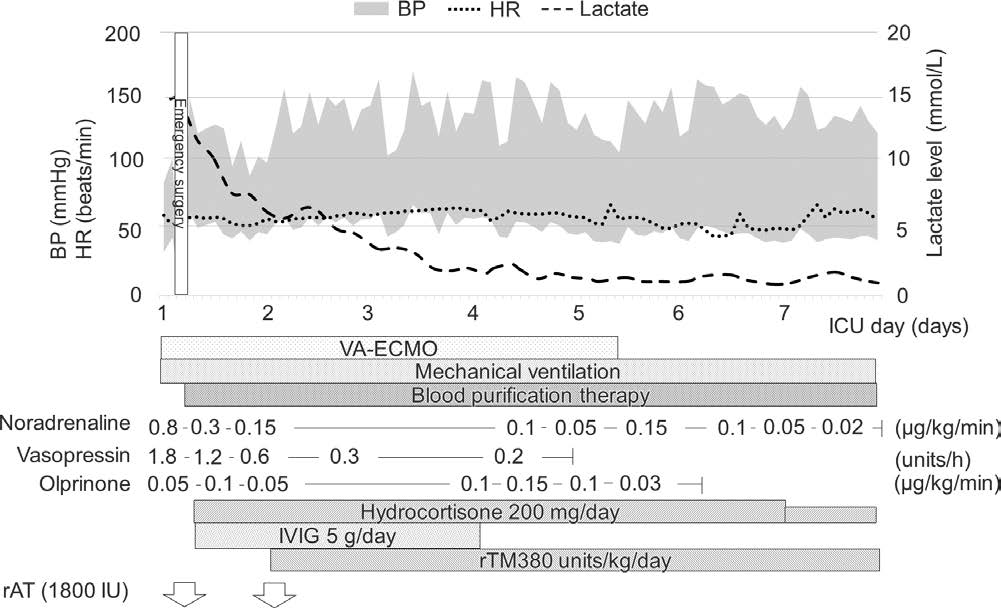
Clinical course and treatment after ICU admission. On admission to the ICU, peripheral hypoperfusion was observed, and the lactate level was 15.0 mmol/L. The patient required noradrenaline (0.8 μg/kg/min) and vasopressin (1.8 units/h) to maintain the perfusion pressure under VA-ECMO support with a blood flow of 3.0 L/min. Because of extreme peripheral hypoperfusion, olprinone was also administered. After emergency surgery under VA-ECMO for duodenal perforation and peritonitis, blood purification, adjunctive hydrocortisone therapy, intravenous immunoglobulin administration, and treatment for disseminated intravascular coagulation (recombinant antithrombin and recombinant soluble thrombomodulin) were initiated, along with antibiotic administration. Thereafter, a rapid improvement in hemodynamics was noted, the lactate levels normalized on day 4 of ICU admission, and the patient was weaned off VA-ECMO on day 5 in the ICU. BP = blood pressure, HR = heart rate, ICU = intensive care unit, IVIG = intravenous immunoglobulin, rTM = recombinant soluble thrombomodulin, rAT = recombinant antithrombin, VA-ECMO = venoarterial extracorporeal membrane oxygenation.

After contrast-enhanced computed tomography was performed, she was admitted to the intensive care unit (ICU); peripheral hypoperfusion was observed, and her lactate level was 15.0 mmol/L. She required noradrenaline (0.8 μg/kg/minutes) and vasopressin (1.8 units/hours) to maintain the perfusion pressure under VA-ECMO support, with a blood flow of 3.0 L/minutes (Fig. 2). Transthoracic echocardiography revealed normal cardiac function with an ejection fraction of 50% and no other cardiac abnormalities, but the patient had extreme peripheral hypoperfusion and was treated with olprinone, a phosphodiesterase 3 inhibitor, started at a low dose of 0.05 μg/kg/minutes. In addition, she had multiple organ failure; the blood biochemistry test results were as follows: white blood cell count, 2000/µL; hemoglobin level, 5.1 g/dL; platelet count, 11,000/µL; prothrombin time-internationally normalized ratio, 4.77; D-dimer level, 94.2 µg/mL; aspartate aminotransferase level, 804 IU/L; alanine aminotransferase level, 255 IU/L; and creatinine level, 2.15 mg/dL.

**Figure 3.**
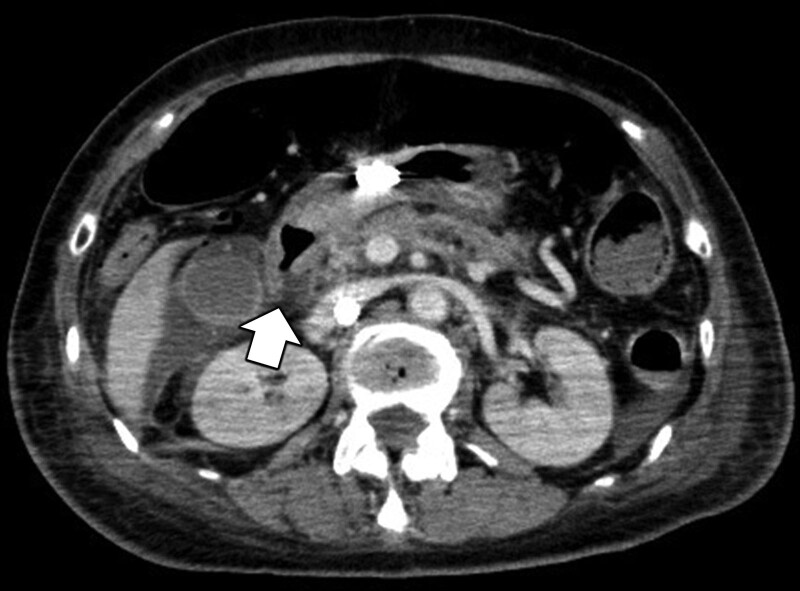
Contrast-enhanced computed tomography scan shows a poor contrast effect in the posterior wall of the duodenal bulb and perforation at this region (white arrow).

Furthermore, contrast-enhanced computed tomography revealed duodenal perforation (Fig. 3); therefore, septic shock owing to peritonitis was diagnosed. Emergency surgery under VA-ECMO support was performed for intraperitoneal lavage and drainage combined with closure of the duodenal perforation. Empirical antibiotic treatment with meropenem, vancomycin, and micafungin was immediately initiated. The patient had progressive multiple organ failure, with a sequential organ failure assessment score of 21 and an acute physiology and chronic health evaluation II score of 40 on day 1 in the ICU. Blood purification therapy with an AN69ST membrane hemofilter (SepXiris 150, Baxter, Tokyo, Japan) and polymyxin B-immobilized fiber column direct hemoperfusion (TORAYMYXIN PMX-20R, Toray Industries, Tokyo, Japan) was initiated to adsorb cytokines and endotoxin. Adjunctive hydrocortisone therapy (200 mg/day), intravenous immunoglobulin administration (5 g/day for 3 days), and treatment for disseminated intravascular coagulation (recombinant antithrombin and recombinant soluble thrombomodulin) were also initiated (Fig. 2). On day 2 in the ICU, clindamycin was also administered to inhibit toxin production because beta-hemolytic streptococci were detected in blood culture test performed on ICU admission and ascites fluid culture test performed during the surgery. We finally diagnosed STSS caused by GBS with duodenal perforation and peritonitis according to the centers for disease control and prevention criteria^[19]^ and recent reports.^[3,4]^ Thereafter, a rapid improvement in hemodynamics was observed; lactate levels normalized on day 4 in the ICU, and the patient was weaned off VA-ECMO on day 5 of ICU admission (Fig. 2). Owing to prolonged disturbance of consciousness, a tracheotomy was performed on day 8 of ICU admission, and she was weaned off the ventilator on day 12 in the ICU. Although she developed candidemia, a cytomegalovirus infection, and severe ICU-acquired weakness, she recovered from multiple organ failure and was transferred to a general ward on day 22 of ICU admission. Eighty-eight days after discharge from the ICU, the patient was transferred for rehabilitation and subsequently underwent radical rectal cancer resection. At the 1-year follow-up, short-term memory impairment and gait disturbance remained, which may have been caused by post-resuscitation encephalopathy. However, she was discharged from the hospital and was under outpatient follow-up.

## 3. Discussion

Here, we report a case of successful treatment of STSS caused by GBS, in which VA-ECMO was introduced for CPA. The patient presented with severe multiple organ failure, including refractory shock after ROSC; however, she rapidly recovered and survived owing to 4 days of ECMO support. The patient was unresponsive to conventional CPR and could not have been saved without ECMO.

Despite the high mortality rate of patients with STSS, specific treatment for the disease has not been established. The rapid progression of circulatory failure owing to STSS leads to refractory shock and finally CPA. Recently, some case reports have demonstrated the effectiveness of VA-ECMO in saving the lives of such patients^[7–13]^; however, its efficacy remains unknown. Therefore, we collected case reports on VA-ECMO for adult patients with STSS and investigated its characteristics and outcomes. The case reports were collected through a search of PubMed using the following search formula: [“Shock, Septic” (medical subject headings [MeSH]) OR “Streptococcal Infections”(MeSH) OR “Fasciitis, Necrotizing”(MeSH) OR “necrotizing soft tissue infection”] AND “Extracorporeal Membrane Oxygenation” [MeSH]. In addition to the centers for disease control and prevention criteria,^[19]^ STSS diagnosis was based on the identification of GAS or non-GAS beta-hemolytic spp., as performed in recent reports.^[3,4,6]^ The cases in which the causative organism was isolated from a sterile site such as blood were classified as “confirmed,” whereas those in which the organism was detected from a non-sterile site were classified as “probable.”

A total of 7 case reports describing 8 patients^[7–13]^ and 1 retrospective observational study describing 10 patients^[15]^ were identified. In addition, 1 case report was manually searched.^[20]^ Therefore, data of 20 patients were examined, including our patient. Table 1 summarizes the characteristics and outcomes of patients with STSS treated using VA-ECMO. The patients were middle aged [53 (41–69) years; presented as median (interquartile range)], and most patients were healthy adults without serious medical conditions. GAS was the causative organism in most cases. When left ventricular failure (LVF) was defined as a left ventricular ejection fraction < 40% as previously reported,^[15]^ 5 (26.3%) patients, including our patient, had no LVF (n = 19; 1 patient was excluded due to missing data). Respiratory status was not detailed in 3 cases (cases 2–4 in Table 1); however, severe respiratory failure was noted in the remaining 17 cases. Severe hyperlactatemia was observed in most cases, and CPR was required in 9 patients (45%) before introducing ECMO. The duration of ECMO was 4.6 (4.0–7.2) days. The overall in-hospital survival rate was 90%. In the analysis that included only cases from an observational study^[15]^ with little concern for publication bias (cases 11–20 in Table 1), the survival rate was also 90%. The survival rate of patients who had CPR before the introduction of ECMO was 89%, while the survival rate of patients without LVF was 80%. The survival rate of patients aged over 60 years was 88%, indicating a high probability of survival. In the analysis of cases of surviving patients whose details are known (cases 1, 2, 4–8, and 10 in Table 1), serious sequelae in major organs such as the heart, lungs, and kidneys were not reported, except for functional impairment owing to limb amputation.

**Table 1:** Characteristics and outcomes of patients with STSS treated using VA-ECMO.

No	Age, years and sex	Underlying condition	Causative pathogen	STSS diagnosis	LVEF, %	Lactate, mmol/L	CPR before ECMO	Duration of ECMO	Outcome	Year^ref^
Case report and this case										
1	18, M	Necrotizing soft tissue infection	*Str. pyogenes*	Confirmed	5–10	-	Yes	5 d	Survived	2010^[3]^
2	39, F	Necrotizing soft tissue infection Post-cesarean delivery	*Str. pyogenes*	Probable	5	9.6	Yes	3 d	Survived	
3	31, M	Exercise-induced rupture of muscle	*Str. pyogenes*	Confirmed	35	-	No	4 d	Survived	2015^[4]^
4	51, M	Chronic leg ulcers Gout	*Str. pyogenes* *St. aureus*	Probable	<10	7.2	No	5 d	Survived	2015^[5]^
5	24, F	Normal pregnancy	*Str. pyogenes*	Confirmed	10	20.0	No	5 d	Survived	2016^[6]^
6	70, M	Gluteal abscess Gout	*Str. pyogenes*	Confirmed	25	-	Yes	3 d	Survived	2018^[7]^
7	41, M	Necrotizing fasciitis	*Str. pyogenes*	Probable	15	4.4	No	4 d	Survived	2018^[8]^
8	31, F	Normal pregnancy	*Str. pyogenes*	Confirmed	57	9.2	Yes	3 d	Survived	2020^[9]^
9	56, M	Post hemorrhoidectomy	*Str. pyogenes*	Confirmed	-	-	Yes	2 h	Died	2021^[19]^
10	60, F	Perforation peritonitis Rectal cancer under chemoradiotherapy	*Str. agalactiae*	Confirmed	50	15.0	Yes	4 d	Survived	This case
Observational study										
11	45, M	Influenza A pneumonia	*Str. pyogenes*	Confirmed	30	13.3	Yes	9 d	Survived	2019^[11]^
12	74, M	Fasciitis Rheumatoid arthritis Asthma	*Str. pyogenes*	Confirmed	40	5.5	No	6.6 d	Survived	
13	69, F	Fasciitis	*Str. pyogenes*	Confirmed	30	11.8	Yes	3.9 d	Survived	
14	73, M	Pneumonia Deep vein thrombosis	*Str. pyogenes*	Confirmed	30	5.0	No	19.1 d	Survived	
15	70, M	Pneumonia Post-cardiovascular surgery	*Str. pyogenes*	Confirmed	25	8.0	No	9 d	Survived	
16	45, F	Influenza B pneumonia	*Str. pyogenes*	Confirmed	60	11.9	No	4 d	Survived	
17	67, F	Fasciitis Hypertension COPD Psoriasis Alcohol abuse	*Str. pyogenes*	Probable	25	4.6	No	4.2 d	Survived	
18	75, F	Pyelonephritis Chronic renal failure Hypothyroidism	Beta-hemolytic streptococci	Confirmed	40	5.2	No	19.4 d	Died	
19	45, M	Influenza A pneumonia Eosinophilic fasciitis	*Str. pyogenes*	Confirmed	37	8.0	No	11.6 d	Survived	
20	55, M	Pneumonia	Beta-hemolytic streptococci	Probable	25	10.0	Yes	5.9 d	Survived	

COPD = chronic obstructive pulmonary disease, CPR = cardiopulmonary resuscitation, F = female, LVEF = left ventricular ejection fraction, M = male, St = Staphylococcus, Str = Streptococcus, STSS = streptococcal toxic shock syndrome, VA-ECMO = venoarterial extracorporeal membrane oxygenation.

VA-ECMO for refractory septic shock in adults is controversial^[21]^; a recent systematic review and meta-analysis reported an in-hospital survival rate of 36.4% (95% confidence interval [CI]: 23.6%–50.1%).^[17]^ The pathophysiology of septic shock typically involves distributive shock without severe abnormalities in cardiac function. VA-ECMO is not effective for managing this, with an in-hospital survival rate of 32.1% (95% CI: 8.7%–60.7%).^[17]^ In contrast, in cases of septic shock with severe LVF (ejection fraction < 20%), in which the highest efficacy is expected, the survival rate was 62.0% (95% CI: 51.6%–72.0%).^[17]^ Besides distributive shock without LVF,^[13]^ age over 60 years^[10]^ and CPA before ECMO induction^[14]^ have been cited as factors that prevent VA-ECMO from improving prognosis; however, differences in efficacy based on bacterial species or type of sepsis have not been examined.

The mortality rate for septic shock is approximately 40%,^[22,23]^ and Kaplan–Meier curves show that this rate is reached relatively slowly over 30 days.^[24]^ The mortality rate of patients with STSS is also 40%,^[1,2]^ although most deaths occur within a few days of onset, with a few patients dying after the fifth day.^[25]^ In other words, the prognosis of STSS may be improved if death within 5 days of onset can be avoided. The use of VA-ECMO for shock that does not respond to conventional treatment or for CPA may be an effective life-saving strategy. In fact, in our literature review, 90% of the patients could be saved with VA-ECMO assistance during the first 4 to 5 days after onset, and similar results were obtained when case reports, which have the possibility of publication bias, were excluded. Surprisingly, similar survival rates were also achieved in patients who required CPR. In addition, the survival rates of patients without LVF were 80%. A recent report showed that even patients with refractory septic shock with distributional shock may have an improved prognosis if VA-ECMO is provided (hospital survival rate of 70.6%).^[15]^ Improved tissue oxygenation by VA-ECMO stabilizes hemodynamics, especially in distributive shock with high lactate levels suggestive of increased anaerobic metabolism.^[15]^ Compared with common septic shock, STSS may have characteristics that make ECMO more effective.

The most important role of VA-ECMO in STSS may be to support organ blood flow and oxygen delivery early in patients with refractory shock or CPA and multiple organ failure, including ARDS, and to provide extra time to treat streptococcal infection. In our case, the patient developed CPA and ARDS and had prominent lactic acidosis with hyperkalemia. The introduction of VA-ECMO may have avoided acute mortality and allowed surgery to remove the source of infection, which may have contributed to saving the life of the patient; without ECMO, the patient could have died.

In recent years, the frequency of STSS has increased, along with the incidence of invasive infections by GAS and other groups of beta-hemolytic streptococci.^[3,26]^ Saving the lives of patients with fatal STSS remains a major concern for health care providers. VA-ECMO for patients with fatal STSS may contribute to an improved prognosis; however, further reporting of cases, database establishment, and validation are needed to prove its efficacy. As a clinical consideration, because the diagnosis of STSS is often not confirmed in the acute phase of disease onset, the decision to introduce VA-ECMO should be based on a comprehensive assessment of the patient’s background and clinical course. If the condition is fatal, VA-ECMO should be introduced as early as possible. In our case, despite prompt CPR, the patient had post-resuscitation encephalopathy; as CPA was caused by distributive shock, chest compressions may have been insufficient to maintain effective perfusion pressure. Additionally, ECMO can lead to bleeding, infection, and mechanical complications^[17]^; therefore, caution should be exercised because the introduction of ECMO in less experienced facilities may lead to a worse prognosis for patients.

## 4. Conclusion

In patients with STSS with rapid progressive refractory shock or CPA, VA-ECMO in the acute phase may improve prognosis by ameliorating tissue oxygenation and providing extra time to treat invasive streptococcal infection. However, further data accumulation and validation are needed to establish efficacy.

## Acknowledgments

We would like to thank Editage (www.editage.jp) for English language editing.

## Author contributions

**Conceptualization:** Naoya Iwasaki, Motohiro Sekino, Tetsuro Tominaga, Takeshi Tanaka, Hiroshi Araki, Rintaro Yano, Sojiro Matsumoto, Taiga Ichinomiya, Ushio Higashijima, Takashi Nonaka, Koichi Izumikawa, Tetsuya Hara.

**Data curation:** Naoya Iwasaki, Motohiro Sekino.

**Formal analysis:** Naoya Iwasaki, Motohiro Sekino, Hiroshi Araki, Rintaro Yano, Sojiro Matsumoto, Taiga Ichinomiya, Ushio Higashijima.

**Investigation:** Naoya Iwasaki, Motohiro Sekino, Tetsuro Tominaga, Takeshi Tanaka, Hiroshi Araki, Rintaro Yano, Sojiro Matsumoto, Taiga Ichinomiya, Ushio Higashijima, Takashi Nonaka, Koichi Izumikawa, Tetsuya Hara.

**Supervision:** Motohiro Sekino, Tetsuya Hara.

**Visualization:** Naoya Iwasaki, Motohiro Sekino.

**Writing – original draft:** Naoya Iwasaki.

**Writing – review & editing:** Motohiro Sekino, Tetsuro Tominaga, Takeshi Tanaka, Hiroshi Araki, Rintaro Yano, Sojiro Matsumoto, Taiga Ichinomiya, Ushio Higashijima, Takashi Nonaka, Koichi Izumikawa, Tetsuya Hara.
